# Glycyrrhizic Acid Mitigates Tripterygium-Glycoside-Tablet-Induced Acute Liver Injury via PKM2 Regulated Oxidative Stress

**DOI:** 10.3390/metabo12111128

**Published:** 2022-11-17

**Authors:** Qixin Wang, Yuwen Huang, Yu Li, Luyun Zhang, Huan Tang, Junzhe Zhang, Guangqing Cheng, Minghong Zhao, Tianming Lu, Qian Zhang, Piao Luo, Yinhua Zhu, Fei Xia, Ying Zhang, Dandan Liu, Chen Wang, Haiyan Li, Chong Qiu, Jigang Wang, Qiuyan Guo

**Affiliations:** 1 Artemisinin Research Center, Institute of Chinese Materia Medica, China Academy of Chinese Medical Sciences, Beijing 100700, China; 2 College of Food Science and Engineering, Bohai University, Jinzhou 121013, China; 3 Institute for History of Chinese Medicine and Medical Literature, China Academy of Chinese Medical Sciences, Beijing 100073, China; 4 School of Medicine, Foshan University, Foshan 528000, China; 5 School of Public Health, Guangxi Medical University, Nanning 530021, China

**Keywords:** activity-based protein profiling, cysteine-specific probe, glycyrrhizic acid, oxidative stress, pyruvate kinase, tripterygium glycoside tablet

## Abstract

Tripterygium glycoside tablet (TGT), as a common clinical drug, can easily cause liver damage due to the narrow therapeutic window. Glycyrrhizic acid (GA) has a hepatoprotective effect, but the characteristics and mechanism of GA’s impact on TGT-induced acute liver injury by regulating oxidative stress remain unelucidated. In this study, TGT-induced acute liver injury models were established in vitro and in vivo. The levels of alanine aminotransferase (ALT), aspartate aminotransferase (AST), alkaline phosphatase (AKP), superoxide dismutase (SOD), malondialdehyde (MDA), glutathione (GSH), catalase (CAT), lactate dehydrogenase (LDH), tumor necrosis factor-α (TNF-α), interleukin-1β (IL-1β) and interleukin-6 (IL-6) were quantified. The anti-apoptotic effect of GA was tested using flow cytometry. Potential target proteins of GA were profiled via activity-based protein profiling (ABPP) using a cysteine-specific (IAA-yne) probe. The results demonstrate that GA markedly decreased the concentrations of ALT, AST, AKP, MDA, LDH, TNF-α, IL-1β and IL-6, whereas those of SOD, GSH and CAT increased. GA could inhibit TGT-induced apoptosis in BRL-3A cells. GA bound directly to the cysteine residue of PKM2. The CETSA and enzyme activity results validate the specific targets identified. GA could mitigate TGT-induced acute liver injury by mediating PKM2, reducing oxidative stress and inflammation and reducing hepatocyte apoptosis.

## 1. Introduction

Tripterygium glycoside tablet (TGT) is a common preparation in Chinese medicine *Tripterygium wilfordii* Hook. f. (TwHF) that can be applied in treating rheumatoid arthritis (RA) and nephrotic syndrome [1]. However, the clinical application of TGT has been limited by adverse reactions; among them, hepatotoxicity has been reported frequently [2].

TGT-induced acute liver injury is a kind of drug-induced liver injury (DILI) with features caused by direct toxicity of drugs or their metabolites [3]. The drugs or their metabolites can act directly on hepatocytes and also cause excessive oxidative stress and inflammation, amplifying hepatocyte damage through apoptosis or necrosis [4,5,6,7,8]. Oxidative stress is the pathophysiological cause of a variety of liver injuries [9]. As a key pathogenic factor of DILI, oxidative stress leads to the progression of liver injury, liver fibrosis and even cirrhosis [10]. Regulating oxidative stress plays an important role in alleviating liver injury [11]. Results show that a number of hepatoprotective drugs, especially Chinese herbal medicine preparations, have significant protective and therapeutic effects on oxidative liver disease through a variety of mechanisms [12,13].

As the main active ingredient of *Glycyrrhiza glabra* L. (licorice), being isolated from the roots and rhizomes, triterpene glycyrrhizic acid (GA) has a detoxifying effect on the liver and is a main bioactive component of anti-inflammatory and anti-viral medications [14]. In our previous study, we discovered that GA effectively reduced the serum alanine aminotransferase (ALT), aspartate aminotransferase (AST), alkaline phosphatase (AKP) and total bilirubin (TBIL) concentrations, increased superoxide dismutase (SOD) and glutathione peroxidase (GSH-Px) and improved liver morphology in TGT-induced acute liver injury mice [15]. However, the direct targets of GA action in combating acute liver injury remain to be elucidated. Thus, we aimed to investigate the liver-protective effect of GA on the base of a TGT-stimulated rat hepatocytes (BRL-3A) model. Potential target proteins of GA were then profiled via activity-based protein profiling (ABPP) using a cysteine probe (IAA-yne). 

## 2. Materials and Methods

### 2.1. Reagents

The glycyrrhizic acid (GA), TGT and GC were the same as those used in our previous study [15]; Biotinazide, TBTA, TCEP, Rhodmine-N_3_ and CuSO_4_ were bought from Sigma (St. Louis, MO, USA). TMT 10plex™ reagent, agarose resin and Trypsin were purchased from Thermo Fisher (Waltham, MA, USA). Specific primary antibodies against PKM2 were purchased from Proteintech (Wuhan, China). GAPDH was obtained from Affinity Biosciences (Cincinnati, OH, USA).

### 2.2. TGT-Induced Acute Liver Injury Mouse Model and Administration

Sixty male (C57BL/6J) mice were obtained from Beijing Vital River Laboratory Animal Technology Co. Ltd. (Beijing, China), animal license no. SYXK (Beijing, China) 2021-0011. After acclimatizing for a period of time, the mice were divided into Con, Mod, GC, GA-Low, Mid and High groups, with 6 mice in each group.

Acute liver injury was established by a single gavage of TGT 270 mg/kg after GA or GC administration for 6 days, and the animals were sacrificed 18 h later. Oral administration of 75 mg/kg GC (positive control) and intraperitoneal injections of 25, 50 and 100 mg/kg GA were administered to the low-, medium- and high-dose groups, the concentrations of which were based on our previous study [15]. The control group received the same treatment without drug administration.

Mice were weighed before sacrifice and the liver, kidney, spleen and thymus were weighed to calculate organ coefficient. Livers were collected for the next experimental validation.

### 2.3. Cell Culture

BRL-3A cells were mailed from Shanghai zhongqiaoxinzhou biotech (Shanghai, China). Cells were cultured in DMEM medium containing 10% FBS (Corning, Inc., Corning, NY, USA) and 1% penicillin–streptomycin (Thermo Fisher Scientific, Waltham, MA, USA) at 37 °C and 5% CO_2_.

When BRL-3A grew to 80–90%, it was treated with TGT active pharmaceutical ingredients, and the IC50 value was calculated to select the model concentration. GA was administered at 1, 5, 10, 20, 40, 60, 80 and 100 μM. The control group was treated with the solvent DMSO. CCK8 assay (Keygen Biotech Co., Ltd., Nanjing, China) was used to detect the effect of drugs on BRL-3A cells.

### 2.4. Cytokine Assays

Blood samples were obtained through the eyeballs of mice, left for 30 min, centrifuged at 3000 rpm for 15 min and then stored at −80 °C for inflammatory cytokine detection. BRL-3A cells were extracted as required by the kit. The serum inflammatory factors TNF-α, IL-1β and IL-6 were detected by the elisa kit (Jiangsu Jingmei Biotechnology Co., Ltd., Jiangsu, China).

### 2.5. Oxidative Stress Indicator Detection

Liver and cell contents of SOD, MDA, GSH, CAT and LDH were analyzed using commercial kits (Nanjing Jiancheng Bioengineering Institute, Nanjing, China) and quantified by a 120 automatic biochemical analyzer (Toshiba Co., Ltd., Shibaura, Japan).

### 2.6. Liver Function Indicator Analysis

The concentrations of ALT, AST and AKP in cell supernatants were detected by the kits (Nanjing Jiancheng Bioengineering Institute, Nanjing, China).

### 2.7. HE and Immunofluorescence Staining

Mouse livers were stored in 4% paraformaldehyde, dehydrated, embedded in paraffin and sliced for HE staining. The pathological changes in the liver were observed by scanning photographs, and the image magnification was 40×.

The liver sections were rounded with PKM2 antibody for 12 h at 4 °C, after washing three times with TBST, and secondary antibodies were added and shaken for 1 h at room temperature on a shaker. TBST was rinsed again, stained with DAPI for 5 min and covered with an anti-fading fixative, and fluorescence images were subsequently captured.

### 2.8. Apoptosis Assay and Detection of Mitochondrial Membrane Potential

The experiment was carried out according to the instructions of the apoptosis detection kit (Beyotime Biotechnology Co., Ltd., Shanghai, China). BRL-3A cells were treated with 60, 80 and 100 μM GA and 25 μg/mL TGT for 24 h. DMSO was used as the control, and Annexin V-FITC/PI double staining was applied to incubate cells in the dark at room temperature for 10 min, flow cytometry (Beckman, Brea, CA, USA) was used to analyze apoptosis.

For mitochondrial membrane potential detection, cells were collected and subjected to JC-1 staining with the working solution, left at 37 °C for 20 min, washed twice with staining buffer and then tested on the flow cytometer.

### 2.9. The Competitive In-Gel Fluorescence Labeling of GA in BRL-3A Cells

BRL-3A cells (5 × 10^5^ cells/well) were grown to 90% confluence in 6 cm plates, incubated with GA (0, 50 and 100 μM) for 4 h and washed three times with PBS. Cells were harvested and sonicated on ice until clear after addition of cell lysates (200 μL RIPA containing 1% protease inhibitor). The protein lysis was obtained by centrifugation at 20,000× *g* for 20 min, and diluted to 1 mg/mL with PBS using a BCA kit. After treatment with different concentrations of GA, 100 μL of cell lysis was selected and labeled with the cysteine-specific probe (IAA-yne) for 1 h in the shaker (800 rpm, 37 °C). Then, 10.1 μL of click buffer was added to each sample, including 6 μL of TBTA (10 mM in DMSO), 2 μL of CuSO_4_ (50 mM in ddH_2_O), 2 μL of TCEP (50 mM in ddH_2_O) and 0.1 μL of TAMRA-azide (50 mM in DMSO), which were incubated in a shaker at 37 °C for 2 h. Then, 1 mL acetone was added into the mixtures to precipitate the labeled proteins at −80 °C for 30 min. The supernatant was discarded by centrifugation for 20 min at 20,000× *g*, and the acetone was evaporated. Proteins were relysed with 40 μL of 1× loading buffer, denatured by metal bath heating at 95 °C for 10 min and finally separated on a 10% SDS-PAGE gel. Fluorescent gel images were captured using an Azure Sapphire RGB NIR scanner (Dublin, CA, USA), and total proteins were stained using Coomassie Brilliant Blue.

### 2.10. The Streamlined Cysteine-Activity-Based Protein Profiling

In order to search for the cellular protein targets based on active GA interactions, we performed competitive property profiling experiments. A new desthiobiotin iodoacetamide (DBIA, ChomiX Biotech Co., Ltd., Nanjing, China) probe with better enrichment than alkyne-iodacetamide was selected for the following steps. They were divided into 50 μM DBIA, 50 μM DBIA + 50 μM GA and 50 μM DBIA + 100 μM GA groups. The cell culture, GA treatment and DBIA labeling procedures were similar to those previously described for in-gel fluorescent labeling of GA in BRL-3A cells. After probe labeling was completed, 5 mM DTT was added and maintained at 25 °C for 30 min to quench excess DBIA and reduce disulfide bonds, and 20 mM IAA was added to alkylate the reduced cysteine residues for 20 min at 25 °C in the dark. Subsequently, proteins were precipitated with a chloroform–methanol–water system (1:4:3), 20,000× *g* for 3 min at 25 °C, and the precipitate was washed twice with 500 μL of methanol to remove excess DBIA. For digestion, 200 μL of 200 mM EPPS (pH 8.52 M) and 2 μL of trypsin and LysC (1 μg/μL, Promega) were added and digested overnight at 37 °C using a ThermoMixer at 800 rpm. For peptide labeling, digested peptides containing DBIA-conjugated cysteines were labeled by a TMT 10plex™ Mass Tag reagent (Thermo Scientific, Waltham, MA, USA) according to the instructions. Next, 0.3% hydroxylamine was used to quench the excess TMT reagent. Then, all TMT-labeled samples were combined and dried with a Speedvac to remove all acetonitrile. A total of 100 μL of streptavidin magnetic beads (Thermo Scientific, Waltham, MA, USA) was added to TMT-labeled pooled samples, dissolved in 1 mL of PBS and rotated for 4 h at room temperature for peptide enrichment. To remove nonspecific bindings, beads were washed according to the following procedure: 1 mL of PBS, 1 mL 0.1% of SDS and 1 mL of dd water, in triplicate. Next, 200 μL of 0.1% Formic acid in 50% acetonitrile was added in a shaker for 30 min at room temperature to elute peptides from the beads, centrifuged and transferred to a new tube. Then, the elution step was repeated, all the cysteine-containing peptides were combined together, and a Speedvac was used for the drying step. Finally, after desalting with 0.1% formic acid, DBIA-labeled peptides were eluted from a C18 column with 0.1% Formic acid-50% acetonitrile, and samples were analyzed with an Orbitrap Fusion Lumos (Thermo Scientific, Waltham, MA, USA).

### 2.11. Protein Purifcation

ThePKM2 DNA Cys424Ser mutant (GenBank Accession NP_002645.3) was cloned to pET28a. Escherichia coli BL21 was used to transfuse the plasmid to express the protein. The bacterial solution was collected, purified with imidazole on a Ni-NTA column and concentrated. The purity of the recombinant proteins was observed using Coomassie Brilliant Blue.

### 2.12. PKM2 Enzyme Determination

The enzymatic activity of purified recombinant PKM2 was detected with and without GA (100 μM) according to the instructions of PK activity detection kit (Solarbio, Beijing, China). We used a microplate reader to scan the activity every 2 min for 22 min.

### 2.13. CETSA Validation

CESTA was used to verify the binding of GA to potential protein targets. The proteins induced by TGT in cells were extracted with ripa and then incubated in equal amounts (1 mg/mL, 1 mL) with GA (100 μM) at room temperature for 1 h. Then, it was divided into 12 aliquots and heated at various temperatures for 6 min, followed by incubation in a thermal cycler at 4 °C for 3 min (Applied Biosystems, Thermo Scientific, Waltham, MA, USA). Proteins were centrifuged at maximum speed for 20 min, precipitated in acetone, redissolved in 1 × loading buffer and boiled at 95 °C for 10 min before WB.

### 2.14. RNA Interference

siRNA sequences (sense 5′-3′ GAUGUCGACCUUCGUGUAAACTT; antisense 3′-5′ GUUUACACGAAGGUCGACAUCTT) targeting PKM2 were designed and synthesized by Sangon (Shanghai, China). Si-PKM2 was transfected into BRL-3A cells using Lipofectamine 2000 (Termo Fisher, Waltham, MA, USA). Transfection results were obtained by WB.

### 2.15. WB Assay

Proteins were extracted from BRL-3A cell lysis, separated by SDS-PAGE gel with different molecular weights and transferred to PVDF membranes. Subsequently, proteins were treated with primary and secondary antibody. The images were visualized with an enzyme-linked chemiluminescence assay (Azure C400 system, Dublin, CA, USA) and analyzed using image J software (version 1.52, Rawak Software Inc., Stuttgart, Germany).

### 2.16. Statistical Analysis

Data were analyzed using GraphPad Prism software (version 8.0, San Diego, CA, USA), and data are presented as mean ± standard deviation (SD). Difference with *p* value less than 0.05 was regarded as statistically significant.

## 3. Results

### 3.1. GA Mitigated Liver Injury by Enhancing Antioxidant Capacity and Inhibiting the Inflammatory Activity in Mice

#### 3.1.1. GA Significantly Altered the Liver Morphology and Organ Index in Mice

**Figure 1:**
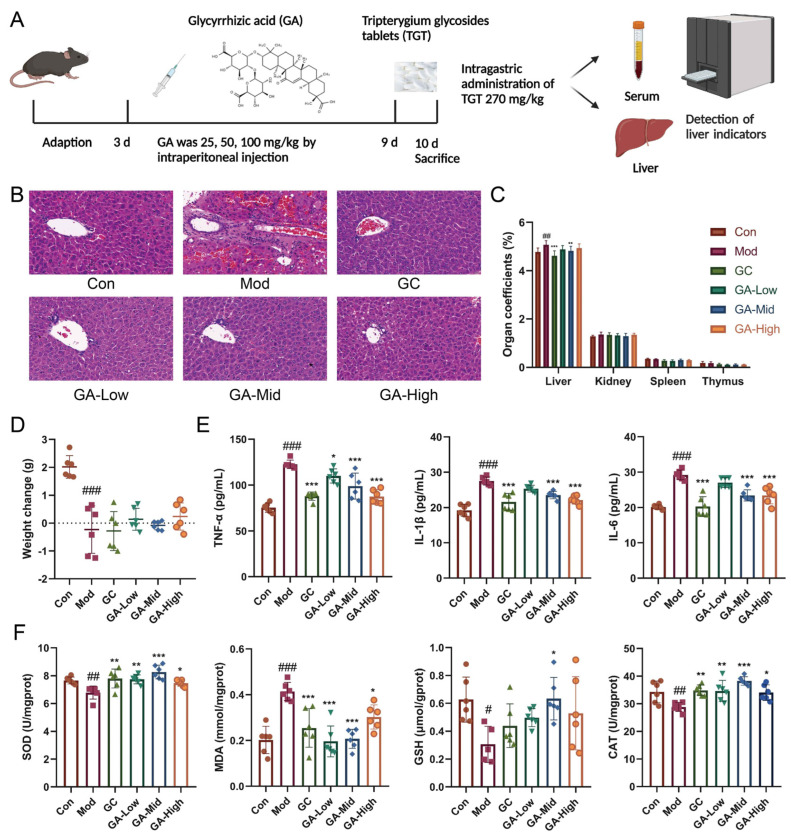
Influence of GA on the weight change, organ index, liver histopathology and biochemical parameters in vivo. (**A**) GA animal experimental protocol; (**B**) representative HE staining of livers (40×) for histological examination. (**C**) The effects of GA on TGT-induced organ coefficient in mice, n = 6; (**D**) effects of GA on weight change in mice, n = 6; (**E**) effects of GA on TNF-α, IL-1β and IL-6 in the serum of mice, n = 6; (**F**) effects of GA on SOD, MDA, GSH and CAT in the liver of mice, n = 6. Data are expressed as the mean ± s; # *p* < 0.05, ## *p* < 0.01, ### *p* < 0.001 vs. control; * *p* < 0.05, ** *p* < 0.01, *** *p* < 0.001 vs. model.

Consistent with our previous and current hematoxylin–eosin (HE) staining results, the Mod group, which was provided with a single oral administration of TGT at a concentration 20 times greater than the clinical equivalent dose, demonstrated obvious inflammatory cell infiltration, steatosis, swelling and congestion of hepatocytes; Ganlixin capsule (GC) and GA treatment significantly improved liver morphology (Figure 1B). Moreover, the organ coefficients demonstrated that TGT significantly increased the liver weight and showed no marked influence on the spleen, kidney or thymus, and the changes in the liver were reversed in the GC and GA-Mid groups (Figure 1C). In terms of changes in body weight, the TGT-treated mice showed a markedly decreased body weight compared with the Con group (Figure 1D).

#### 3.1.2. GA Significantly Increased the Antioxidant Capacity and Reduced the Level of Inflammatory Cytokines in Mice

Through the detection of serum inflammatory cytokines and liver oxidative stress indicators, we found that compared with normal mice, the levels of tumor necrosis factor-α (TNF-α), interleukin-1β (IL-1β) and interleukin-6 (IL-6) of mice in the Mod group were increased, while GC and GA reduced these indicators, and the ratio of the GC, GA-Mid and High group dosage to the Mod group dosage was statistically different (Figure 1E). The contents of SOD, glutathione (GSH) and catalase (CAT) in Mod mice were markedly decreased, while malondialdehyde (MDA) increased; GA significantly increased the content of antioxidant enzymes, while it decreased the level of the oxidative metabolite MDA, whereas GC had no significant effect on GSH (Figure 1F).

### 3.2. GA Improved Hepatocyte Function by Inhibiting TGT-Induced Inflammation and Oxidative Damage In Vitro

#### 3.2.1. GA Treatment Simultaneously Reduced TGT-Induced Hepatotoxicity In Vitro

**Figure 2:**
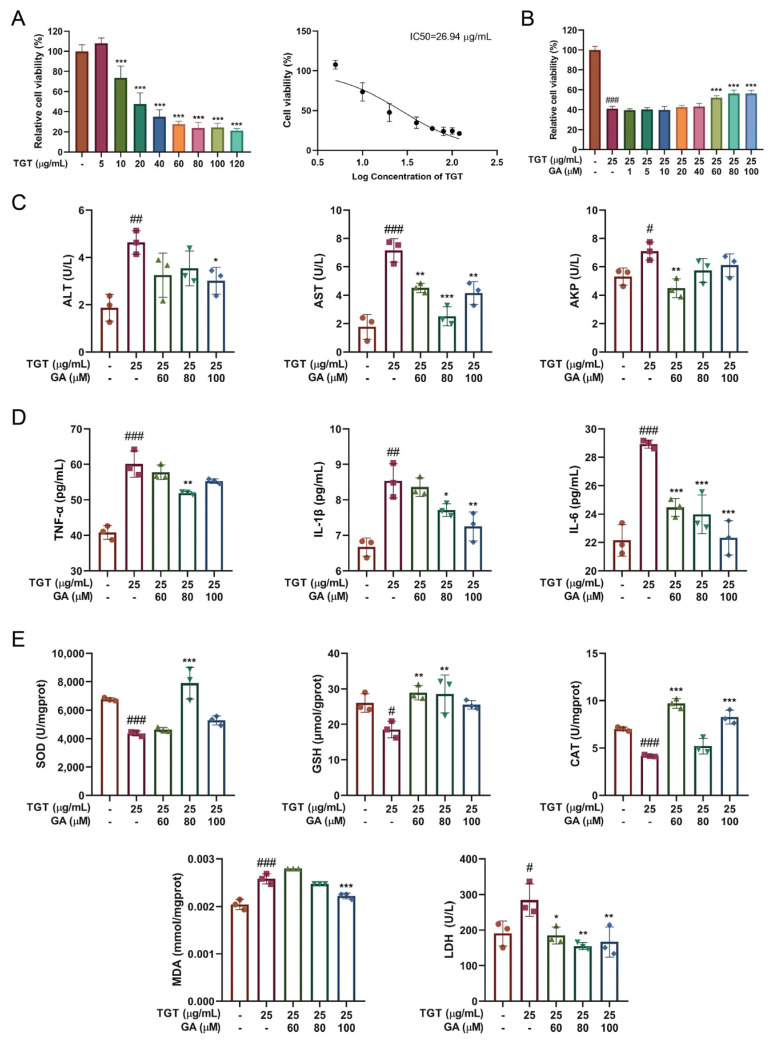
Influence of GA on liver function, oxidative stress indicators and inflammatory cytokines in vitro. (**A**) Effects of TGT on the viability of BRL-3A cells, n = 3; (**B**) influence of GA on the viability of BRL-3A cells, n = 3; (**C**) effects of GA on ALT, AST and AKP in the supernatant of BRL-3A cells, n = 3; (**D**) effects of GA on release of inflammatory cytokines TNF-α, IL-1β and IL-6, n = 3; (**E**) effects of GA on SOD, GSH, CAT, MDA and LDH. Data are expressed as the mean ± s; # *p* < 0.05, ## *p* < 0.01, ### *p* < 0.001 vs. control; * *p* < 0.05, ** *p* < 0.01, *** *p* < 0.001 vs. model.

The hepatocyte toxicity of TGT was measured based on BRL-3A, and unlike in our previous study, TGT active pharmaceutical ingredients instead of the solution of commercial TGT were used in this study. Here, we used 25 μg/mL of TGT IC50 as the model concentration (Figure 2A). The GA concentrations of 1, 5, 10, 20, 40, 60, 80 and 100 μM were applied according to our previous study [15] for subsequent tests. Our results show that when BRL-3A cells were treated with both GA and TGT and cultured for 24 h, GA at 60, 80 and 100 μM significantly attenuated the inhibitory effect of TGT on BRL-3A activity (Figure 2B).

#### 3.2.2. GA Effectively Alleviated TGT-Induced Acute Liver Injury In Vitro

Figure 2C shows that compared with the control group, the levels of ALT, AST and ALP in the TGT group were significantly increased, while those in the GA group were decreased, but there was no significant dose dependence. The supernatant concentrations of MDA, dehydrogenase (LDH), TNF-α, IL-1β and IL-6 in TGT model were abnormally increased, while SOD, GSH and CAT decreased, and GA significantly increased antioxidant enzyme levels, reduced the accumulation of oxidative metabolites and decreased inflammatory cytokines (Figure 2D,E).

### 3.3. GA Promoted Proliferation and Reduced Apoptosis of BRL-3A Cells

#### 3.3.1. GA Effectively Reduced TGT-Induced Hepatocyte Apoptosis

**Figure 3:**
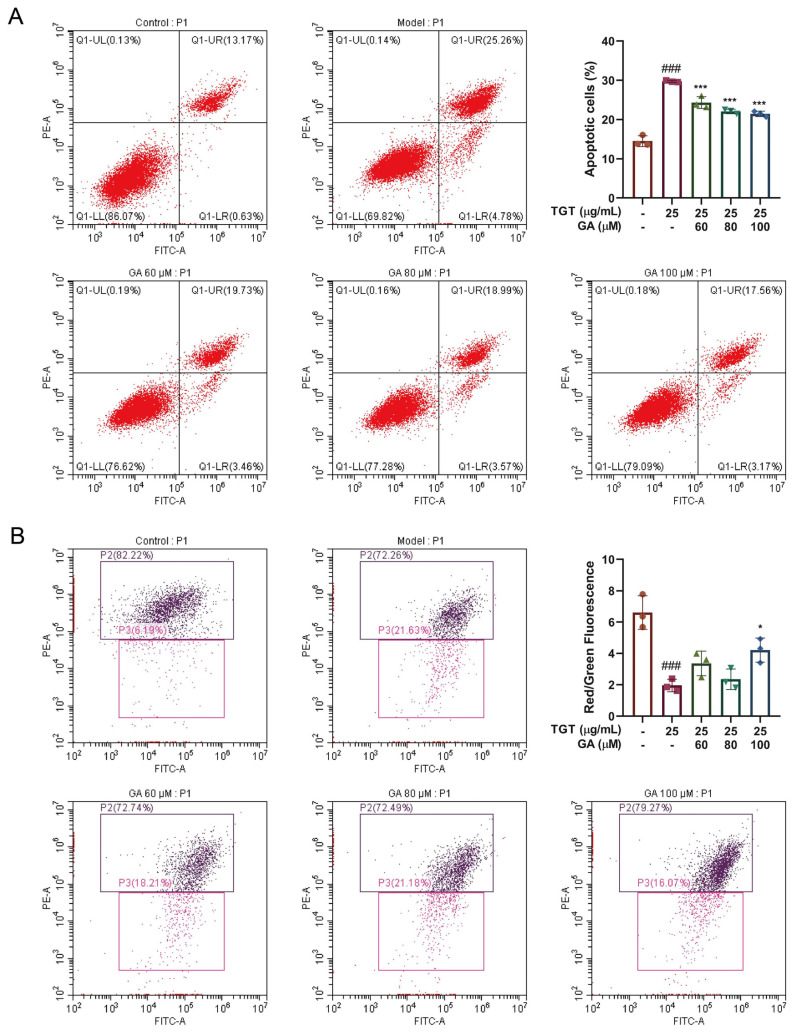
(**A**) Detection of TGT-induced apoptosis with or without GA and the quantitation of apoptosis rates, n = 3; (**B**) detection of TGT-induced decrease in mitochondrial membrane potential with or without GA by flow cytometry and the ratio of red to green fluorescence, n = 3. Data are expressed as the mean ± s; ### *p* < 0.001 vs. control; * *p* < 0.05, *** *p* < 0.001 vs. model.

Flow scatter plots of apoptosis tested by Annexin V-FITC/PI are shown in Figure 3A. The results show that the apoptosis rate of the model group was higher than that of the control group; the number of both early apoptosis and late apoptosis cells was significantly increased. The number of late apoptosis cells in the GA groups was significantly lower than that in the TGT-treated model group. The ratio of cells in different quadrants was calculated to indicate the apoptosis of cells. Compared with the non-intervention control group, the apoptosis rate was significantly increased in the model group. GA plays a protective role in hepatocytes by reducing apoptosis.

#### 3.3.2. GA Reversed the TGT-Induced Increase in Mitochondrial Membrane Potential

The reduction in mitochondrial membrane potential is regarded as a hallmark event during the early stage of apoptosis. Flow cytometry was used to test the mitochondrial membrane potential and the anti-apoptotic effect of GA. This is shown in the scatter plot in Figure 3B. Mitochondrial membrane potential was significantly reduced in the model group. Compared with the control group, the mitochondrial membrane potential was significantly decreased in the model group. On the contrary, the GA-High group had significantly increased potential. The ratio of red to green fluorescence was used for statistics.

### 3.4. GA Bound Directly to PKM2

#### 3.4.1. Fluorescence Labeling and ABPP Identification of GA Targets

**Figure 4:**
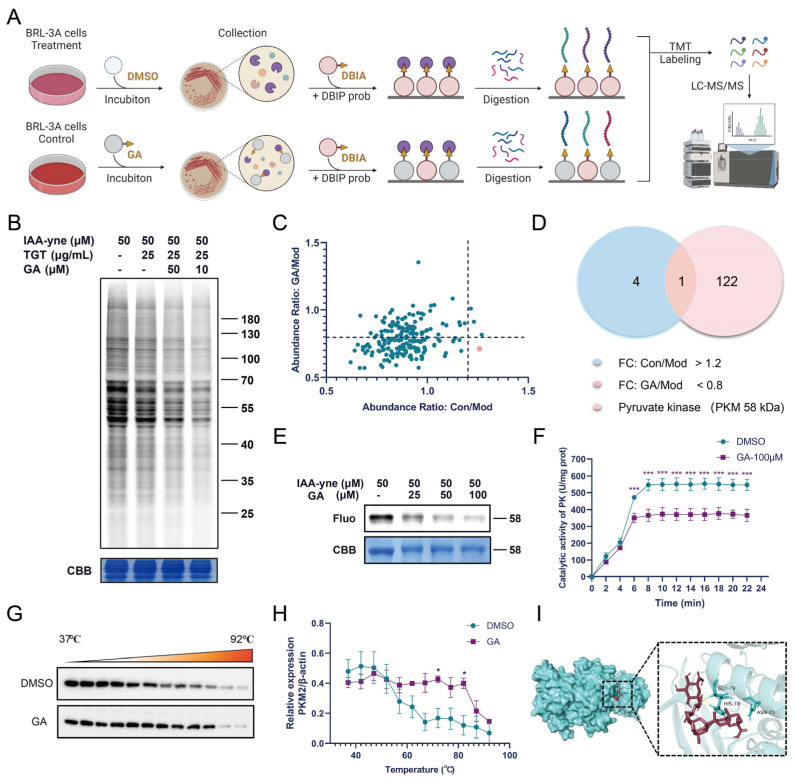
Identification of potential targets of GA in vitro. (**A**) Overall flow of ABPP analysis of GA targets; (**B**) the competition between proteins labeled with 50 μM IAA-yne probe by GA in situ; (**C**,**D**) competitive effects of GA on the binding of PKM to IAA-yne probe by LC-MS/MS analysis; (**E**) GA competes with IAA-yne for binding to purified recombinant PKM2 protein in the in-gel fluorescence assay; (**F**) changes in PK with or without GA treatment, n = 3, *** *p* < 0.001 vs. DMSO; (**G**,**H**) CETSA-WB verification of GA binding to PKM2, n = 3, * *p* < 0.05 vs. DMSO; (**I**) molecular docking simulation of GA binding to PKM2 protein.

ABPP was employed for the protein target identification of GA in BRL-3A cells. The fluorescent gel results show that GA could compete for the fluorescence signal of the protein labeled with the IAA-yne probe, which proved that GA bound to the cysteine residue of the target protein. Differential proteins in different groups were analyzed using mass spectrometry. Pyruvate kinase M (PKM) was selected from the binding proteins of GA according to the abundance ratio for subsequent studies (Figure 4C,D); compared with the Con group, PKM was significantly higher in the Mod group (abundance ratio: Con/Mod > 1.2); compared with the Mod group, PKM was significantly lower in the GA group (GA/Mod < 0.8). Therefore, we turned our attention to PKM in our subsequent analysis.

#### 3.4.2. GA Directly Targeted PKM2 in Activated BRL-3A Cells

As the rate-limiting enzyme of cell glycolysis, pyruvate kinase (PK) has different isoforms [16]. PKM2 has been widely studied in liver fibrosis and liver cancer, so we focused on PKM2 for subsequent validation [17,18]. The purified recombinant PKM2 protein was labeled with fluorescence and its enzymatic activity was detected. Detection with purified recombinant PKM2 protein was consistent with the above results (Figure 4E). Additionally, GA could inhibit the activity of PKM2 in in vitro experiments (Figure 4F). The results of the cellular thermal shift assay−Western blot (CETSA-WB) show that compared with the DMSO group, GA administration reduced PKM2 expression in BRL-3A cells at the beginning, but when combined with PKM2, it increased the thermal stability (Figure 4G,H). These results confirm that PKM2 is the target of GA. In addition, molecular docking simulation analysis of the binding sites of GA and PKM2 showed that GA binds to the GLY-79, HIS-78 and ASN-75 residue of PKM2 (binding energy: −5.21, Figure 4I), which requires further experimental confirmation.

### 3.5. Silencing of PKM2 Inhibited Inflammation in BRL-3A Cells

**Figure 5:**
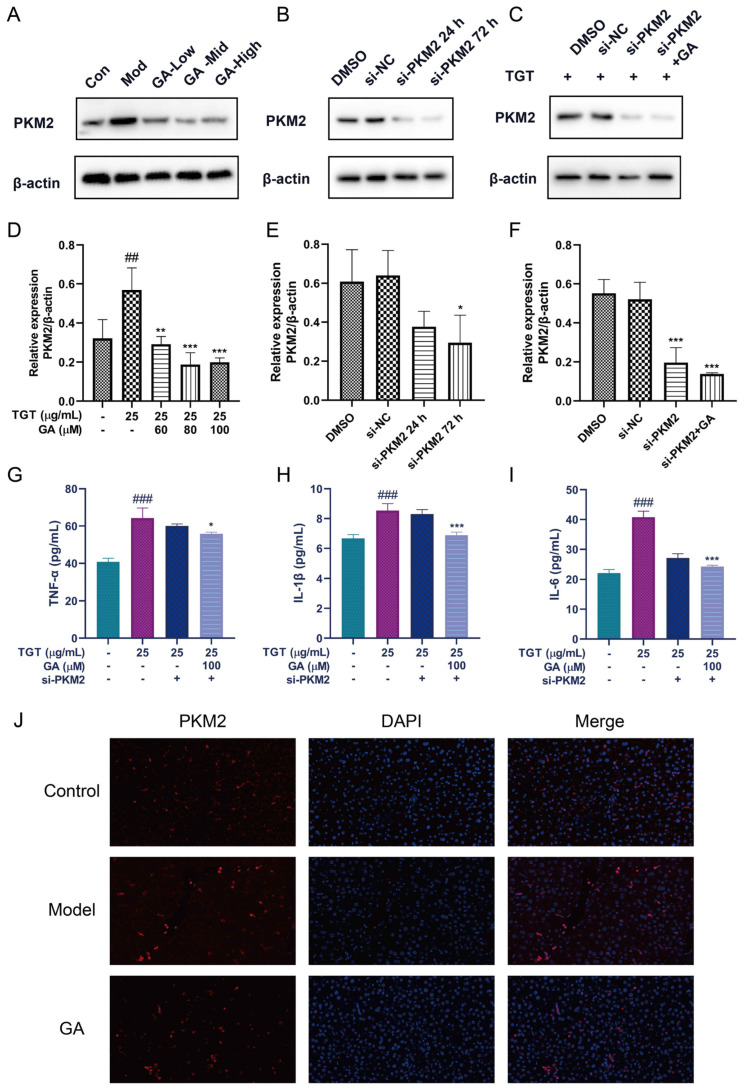
Silencing of PKM2 could inhibit inflammation. (**A**) The expression of PKM2 was detected by WB; (**B**) cells transfected with siRNA for 24 h or 72 h (si-PKM2); (**C**) TGT-induced cells transfected with si-PKM2 72 h with or without GA; (**D**–**F**) the results were analyzed by WB. Data are expressed as the mean ± s; ## *p* < 0.01, ### *p* < 0.001 vs. control; * *p* < 0.05, ** *p* < 0.01, *** *p* < 0.001 vs. model or DMSO, n = 3; (**G**–**I**) the levels of TNF-α, IL-1β and IL-6 in si-PKM2-transfected cells treated with TGT or GA, n = 3; (**J**) localization of PKM2 (red) verified by immunofluorescence staining (40×).

The WB results show that TGT could increase the level of PKM2, and different concentrations of GA could reduce the expression of PKM2 (Figure 5A). The small interfering RNA (siRNA) was transfected into BRL-3A cells to knock down PKM2, and the best effect was achieved at 72 h (Figure 5B). Interference with PKM2 in TGT-induced BRL-3A cells resulted in decreased PKM2 expression; meanwhile, PKM2 expression in TGT-induced BRL-3A cells was further decreased by GA treatment (Figure 5C). In addition, GA combined with si-PKM2 further reduced the expression of inflammatory factors in TGT-induced BRL-3A cells (Figure 5G–I). These results indicate that GA-inhibited PKM2 mainly suppresses the mediated inflammation. Immunofluorescence staining results again demonstrate the high expression of PKM2 in the TGT-treated group, which was reduced after GA treatment (Figure 5J).

## 4. Discussion

TGT, as a common clinical drug for RA [19], has a narrow therapeutic window; thus, it is necessary to explore a solution for TGT-induced liver injury. Licorice can be effectively combined with drugs such as TwHF to attenuate toxicity and enhance efficacy [20,21]. GA, the major active component of licorice, has been reported to significantly improve various indicators of liver function in hepatitis patients [22].

In our previous paper, we discovered GA can significantly change the liver injury state of TGT-mediated acute liver injury in mice. Here, we further validated that the hepatoprotective effect of GA can be realized through increasing the antioxidant capacity and inhibiting inflammation both in vivo and in vitro. Histopathological observation of the liver showed that TGT induced obvious steatosis and inflammatory cell infiltration, and the basic structure of liver cells was obviously damaged. Therefore, we detected the key indicators of oxidative stress in the liver and the levels of inflammatory factors in the serum of mice.

To elucidate the mechanism of GA efficacy, BRL-3A cells were used for the further efficacy validation of GA, which was consistent with the in vivo results. Potential target proteins of GA were then profiled via ABPP using an IAA-yne probe. ABPP has been established as a convenient proteomics tool for detecting the activity and function of proteins in cells. It is often used to study the target profile of small molecules [23]. ABPP can be applied in target identification of bioactive molecules, which has been developed as a powerful technology in drug discovery [24]. Here, ABPP was used to confirm that GA could bind directly to PKM2 in TGT-induced BRL-3A cells, and to clarify how the regulation process of GA inhibits damaging inflammatory responses and oxidative stress, helping to sustain the stability of the antioxidant system. Furthermore, the enzymatic activity and CETSA experiments showed that GA inhibited the activities of PKM2 and its thermostability was improved after GA bound to PKM2. These data confirm that GA directly acted on PKM2.

As a rate-limiting enzyme in the glycolysis pathway, PKM2 regulates liver glycogen metabolism [25]. A previous study showed that increased PKM2 levels and activity aggravate oxidative stress and the inflammatory response [26]. Inhibition of PKM2 shifts glucose catabolism to the pentose phosphate pathway, which protects against redox stress via oxidizing glucose 6-phosphate to produce NADPH and regulating the conversion of hydrogen peroxide to water by reducing glutathione disulfide (GSSG) to GSH [27,28]. In addition, LDH catalyzes the dehydrogenesis of pyruvate to lactate by consuming one molecule of NADH during anaerobic glycolysis. LDH activity can be used to evaluate the degree of cell damage and apoptosis. PKM2 links glycolysis and oxidative stress. Here, we found that GA could effectively alleviate the symptoms of model mice by increasing the SOD, GSH and CAT levels, while also reducing the MDA and LDH content of mouse liver tissues and BRL-3A cells, further attenuating oxidative stress in the liver when subjected to a drug attack.

Furthermore, the depletion of GSH in the body due to lipid peroxidation leads to the production of reactive oxygen species (ROS), which subsequently exacerbates oxidative stress. The immediate oxidative stress was markedly increased by the secretion of various cytokines. ROS and cell debris trigger the release of inflammatory mediators in DILI [29]. Dimeric PKM2 has been reported to promote inflammatory responses by phosphorylating tyrosine residues in STAT3 [30]. In our study, we observed the accumulation of inflammatory cells and the elevation of serum inflammatory cytokines TNF-α, IL-1β and IL-6 after TGT stimulation, and GA had a significant inhibitory effect on inflammation. In addition, the decrease in Annexin V-FITC/PI-positive cells and the increase in mitochondrial membrane potential after GA treatment indicated the protective effect of GA against TGT-induced hepatocyte apoptosis. Therefore, GA may inhibit oxidative stress by acting on PKM2, thereby attenuating the inflammatory response of TGT. Consistent with the above results, we observed a slight down-regulation of TNF-α and IL-1β and a significant down-regulation of IL-6 after PKM2 knockdown.

In summary, our results reveal that GA protects against TGT-induced liver injury through anti-oxidation, anti-inflammation and anti-apoptosis, and thus we expect it to become an alternative treatment for TGT-induced DILI.

## 5. Conclusions

The animal and cell models of TGT-induced acute liver injury were established to elucidate the liver-protective effect and protein targets of GA. GA can significantly reverse drug-induced liver injury via targeting PKM2 to inhibit oxidative stress and the release of pro-inflammatory cytokines, thereby reducing hepatocyte apoptosis. Further research on the mechanism of GA should be conducted, so that GA can be used in clinical practice to combat drug-induced liver injury.

## Data Availability

The data presented in this study are available in the article.
